# Genome‐wide association study for yield and quality of granulated cassava processed product

**DOI:** 10.1002/tpg2.20469

**Published:** 2024-06-16

**Authors:** Cynthia Idhigu Aghogho, Siraj Ismail Kayondo, Saviour J. Y. Eleblu, Adenike Ige, Isaac Asante, Samuel K. Offei, Elizabeth Parkes, Chiedozie Egesi, Edwige Gaby Nkouaya Mbanjo, Trushar Shah, Peter Kulakow, Ismail Y. Rabbi

**Affiliations:** ^1^ West Africa Centre for Crop Improvement (WACCI), College of Basic and Applied Sciences University of Ghana Legon Ghana; ^2^ International Institute of Tropical Agriculture (IITA) Ibadan Nigeria; ^3^ International Institute of Tropical Agriculture (IITA), Eastern Africa Hub Dar es Salaam Tanzania; ^4^ Department of Agronomy and Plant Genetics University of Minnesota Minneapolis Minnesota USA; ^5^ National Root Crops Research Institute Umuahia Nigeria; ^6^ Plant Breeding and Genetics Section, School of Integrative Plant Science, College of Agriculture and Life Sciences Cornell University Ithaca New York USA; ^7^ International Institute of Tropical Agriculture (IITA), c/o ILRI Nairobi Kenya

## Abstract

The starchy storage roots of cassava are commonly processed into a variety of products, including cassava granulated processed products (gari). The commercial value of cassava roots depends on the yield and quality of processed products, directly influencing the acceptance of new varieties by farmers, processors, and consumers. This study aims to estimate genetic advance through phenotypic selection and identify genomic regions associated and candidate genes linked with gari yield and quality. Higher single nucleotide polymorphism (SNP)‐based heritability estimates compared to broad‐sense heritability estimates were observed for most traits highlighting the influence of genetic factors on observed variation. Using genome‐wide association analysis of 188 clones, genotyped using 53,150 genome‐wide SNPs, nine SNPs located on seven chromosomes were significantly associated with peel loss, gari yield, color parameters for gari and eba, bulk density, swelling index, and textural properties of eba. Future research will focus on validating and understanding the functions of identified genes and their influence on gari yield and quality traits.

## Core Ideas

- This study is the first comprehensive genome‐wide association study (GWAS) to uncover the genetic factors influencing gari quality in cassava.
- GWAS identified nine significant single nucleotide polymorphisms (SNPs) closely linked to variations in the measured traits.
- Five core candidate genes and hypothetical proteins were identified, co‐localized with significant SNPs.
- Phosphoglucosamine mutase family protein and the pectin lyase‐like superfamily protein demonstrated associations with swelling index. These findings underscore the potential of molecular breeding to enhance gari yield and quality.

**Abbreviations**
BBX: B‐box
BLINK: Bayesian information and linkage disequilibrium iteratively nested keyway
BLUP: best linear unbiased prediction
CMLM: compressed mixed linear model
GA: genetic advance
GAM: genetic advance as percentage of mean
GCV: genetic coefficient of variation
GWAS: genome‐wide association study
IITA: International Institute of Tropical Agriculture
LD: linkage disequilibrium
LRR: leucine‐rich repeat
MAF: minor allele frequency
PCV: phenotypic coefficient of variation
PEL: pectin lyase‐like
PGM: phosphoglucosamine mutase
QTL: quantitative trait locus
QTN: quantitative trait nucleotides
SNP: single nucleotide polymorphism
TASSEL: Trait Analysis by Association, Evolution, and Linkage
UBC: ubiquiting‐conjugating
VIF: variance inflation factor

## 1 INTRODUCTION

Over half a billion people in Africa, Asia, and Latin America depend on cassava (*Manihot esculenta* Crantz), a clonally propagated root crop for dietary calorie sources (Montagnac et al., 2009). Its remarkable resilience to climate change and poor soil conditions has made it a valuable crop for farmers, processors, and consumers (Burns et al., 2010). It is a versatile crop with the potential for food, industrial (starch and biofuel), and export (chips) applications. Africa primarily produces cassava for human consumption, accounting for over 90% of the total production, whereas in Asia and South America, human consumption takes about 50% and 43%, respectively (Nweke, 2004). Cassava roots, being highly perishable and containing cyanogenic glucosides, are commonly processed into various granulated and paste products for detoxification, increase in shelf‐life, and value addition. By far, the two processed cassava products account for the largest proportion of cassava food consumption in Africa, absorbing more than 90% of the produced roots (Nweke, 2004; Phillips et al., 2004). These products not only play a crucial role in food security but also contribute significantly to local economies by providing employment in both agriculture and food processing industries, thereby offering sustainable income opportunities for smallholder farmers.

The granulated cassava product also known as gari in West Africa and farinha in Brazil is made through grating, fermenting, dewatering, and toasting of semi‐dried mash on dry heat (Udoro et al., 2014). Gari can be eaten dry or soaked in water or milk (akin to breakfast cereals) or made into eba, a West African stiff dough enjoyed with soup (Oduro et al., 2000).

Under optimized processing conditions, genetic variations in gari yield, quantified as the amount of gari produced per hectare (t/ha) or as a percentage (%) relative to the initial weight of the storage roots, can be observed among different varieties (Almazan, 1992; Bassey, 2018; Ibe & Ezedinma, 1981). The yield of gari from a particular cassava variety has significant economic implications, given the fixed costs involved in processing, such as labor, time, energy, and transportation (Ibekwe et al., 2012; Nweke, 2004; Wossen et al., 2017). Besides gari yield, other quality parameters of the processed products are also important and many of these are determined both by varietal (genetic) differences and processing methods (Ngoualem Kégah & Ndjouenkeu, 2023).

Cassava gari quality is one of the most important factors that processors and consumers consider when choosing a variety. Some determinants of gari quality include the color and functional properties such as bulk density and swelling index (Ndjouenkeu et al., 2021; Ngoualem Kégah & Ndjouenkeu, 2023; Teeken et al., 2021). Bulk density is an indicator of the weight of gari and can affect its packaging and distribution. The swelling index is used to evaluate the expansion of gari particles in the presence of cold water. The swelling of gari influences its texture, taste, ease of preparation, digestibility, satiation, and overall consumer satisfaction (Oluwamukomi & Lawal, 2020). Two sensory attributes, including hardness and gumminess measured using the compression test, are important determinants of gari acceptance by consumers (Teeken et al., 2021). Hardness describes the softness or firmness of a product, while gumminess is a secondary parameter of cohesiveness or stickiness (Olaosebikan et al., 2023).

With cassava steadily gaining popularity worldwide and across Africa and a shift toward a demand‐led breeding approach, breeding programs are increasingly focusing on product quality traits in addition to yield. This strategic shift is essential to ensure the adoption of new varieties that are not only high‐yielding, pest‐ and disease‐resistant but also meet the specific quality demands of consumers and processors (Bechoff et al., 2018; Ndjouenkeu et al., 2021; Teeken et al., 2021; Wossen et al., 2017). Traditionally, breeders have faced significant challenges in addressing quality traits in cassava due to several factors. The large population sizes and the limited number of roots available in early breeding stages hinder effective phenotypic selection (Ceballos et al., 2015). Furthermore, the complexity of converting fresh roots to gari and the heterogeneity in processing methods complicate the assessment of quality traits (Abass et al., 2013). These challenges are compounded by the inherently long breeding cycle of cassava, which further delays the realization of improvements in product quality through conventional breeding methods. Despite these challenges, the potential benefits of advanced molecular breeding tools are substantial. These tools offer the possibility to significantly accelerate the development of new cassava varieties that meet specific quality parameters, thereby overcoming traditional limitations and enhancing breeding efficiency.

Among cutting‐edge tools, genome‐wide association studies (GWASs), employing high‐density genetic markers, have proven to be a powerful approach for uncovering genomic regions underlying variation in traits of interest (Wallace et al., 2014). The molecular markers identified through GWAS are instrumental in facilitating marker‐assisted selection, significantly enhancing breeding efficiencies. For cassava, these markers are especially valuable as they allow breeders to make selections during the early testing stages, where a large number of entries are typically evaluated. This approach not only increases selection intensity but also shortens the breeding cycle and reduces the need to manage and process large numbers of plots, thereby significantly cutting costs.

Several studies have successfully utilized GWAS to uncover quantitative trait loci (QTLs) associated with a variety of traits in cassava, including resistance to cassava brown streak disease (Kayondo et al., 2018; Nandudu et al., 2023), cassava root mealiness (Uchendu et al., 2021), cassava green mite pest (Ezenwaka et al., 2018), and cassava root rot (Hohenfeld et al., 2022), as well as dry matter content, provitamin A carotenoid content (Rabbi et al., 2017), and starch pasting properties (Phumichai et al., 2022). These studies highlight the power of GWAS in providing valuable genetic insights for developing cassava varieties with superior agronomic and nutritional traits. QTL discovery and candidate gene identification studies have been conducted for end‐use quality traits in other crops like wheat (*Triticum aestivum*) and rice (*Oryza sativa*). Specific genomic regions associated with flour yield and dough characteristics have been identified in wheat (Chang et al., 2022; Ji et al., 2021; Kristensen et al., 2018, 2019). In durum wheat, multiple chromosomes have been linked to semolina dough color, yield, and firmness (Johnson et al., 2019). In rice, Misra et al. (2018) identified multiple major and minor quantitative trait nucleotides (QTN) linked to traits like hardness, adhesiveness, and springiness.

The objective of this study was to identify specific genomic regions and candidate genes associated with gari yield and quality in cassava, using GWASs and estimate the genetic progress achievable through phenotypic selection.

**Core Ideas**

- This study is the first comprehensive genome‐wide association study (GWAS) to uncover the genetic factors influencing gari quality in cassava.
- GWAS identified nine significant single nucleotide polymorphisms (SNPs) closely linked to variations in the measured traits.
- Five core candidate genes and hypothetical proteins were identified, co‐localized with significant SNPs.
- Phosphoglucosamine mutase family protein and the pectin lyase‐like superfamily protein demonstrated associations with swelling index. These findings underscore the potential of molecular breeding to enhance gari yield and quality.

## 2 MATERIALS AND METHODS

### 2.1 Plant material and field trials

An association mapping panel of 200 cassava clones sourced from the International Institute of Tropical Agriculture (IITA), Oyo, Nigeria, was used in this study. The selection of genotypes was based on their fresh root weight in previous seasons as well as their pedigree and single nucleotide polymorphism (SNP) relationships (https://cassavabase.org/breeders/trial/6630). The genotypes were carefully selected from cycle 4 breeding of the Next Generation Cassava Breeding Project (www.nextgencassava.org) and comprised 119 distinct families derived from biparental crosses involving 63 female and 58 male parents (Table S1).

The collection was established in Ikenne in the first planting season (2020/2021) and Ikenne and Ibadan in the second season (2021/2022). Ikenne is a humid forest located at 6°84′N and 3°69′E, while Ibadan is a derived savanna located at 7°49′N and 3°90′E. The trials were established in October of each year and harvested in October of the following year. The field trials were planted in an augmented design, with each block comprising three replicated standard checks. In each block, a total of 25 genotypes were included, with the three standard checks randomly distributed within the block. The selection of the three common standard checks, namely, TMEB 419, TMS‐IBA000070, and TMS30572, was based on their stability and consistent product yield (Aghogho et al., 2022). Each plot had dimensions of 4 m by 5 m and consisted of 20 plants established in four rows, with a spacing of 1 m between rows and 0.8 meters within rows. Healthy stem cuttings measuring 20–25 cm in length were used as planting material. Regular manual weeding was performed throughout the experimental period to maintain a clean field.

### 2.2 Gari processing

Ten kilograms of cassava roots harvested 12 months after planting were processed into gari at the IITA research facility, following the established standard operating procedure (Abass et al., 2013). The roots were peeled, washed, drained, and mechanically grated (Dandrea Agriport Grater, Maquinas d' Andrea). The resulting grated mash was then placed into jute bags and allowed to ferment naturally at room temperature for 72 h. The fermented mash was subsequently dewatered using a hydraulic press for 24 h and the resulting pressed cake was pulverized and sieved to eliminate any fibrous materials. The semi‐dry product was subsequently toasted into gari in a stainless‐steel pan at a temperature of 120.5°C until it reached a moisture content ranging between 8% and 10%. Once the gari had cooled down, it was carefully stored in a cool dry place in airtight containers until further quality analysis. Strict monitoring was carried out at each processing step to minimize operator variation and reduce sample batch effects.

### 2.3 Eba preparation

Eba, the stiff dough, was prepared in duplicate from 100 g of gari samples according to the approved standard operating procedure of the RTB foods (https://doi.org/10.18167/agritrop/00604). Gari was gradually added into 250 mL of hot water in a plastic bowl, which was covered and allowed to stand for 60 s to enable gelatinization. After this initial step, the gari‐water mixture was transferred to a 30 L planetary dough mixer set at 142 rpm and mixed for another 60 s to form a stiff dough. The freshly prepared eba was immediately scooped into aluminum foil, wrapped tightly to prevent moisture loss, and placed in a styrofoam box to maintain warmth until quality assessment. The temperature of the boiling water and the dough was consistently monitored using a thermometer to ensure accuracy in replication.

### 2.4 Product quality trait phenotyping

The association panel was phenotyped for selected traits based on their influence on either productivity or consumer acceptability. The productivity‐related traits included peel loss and yield of gari (expressed as a percentage), which are critical for evaluating the efficiency and output of the production process. Consumer‐oriented traits were color parameters for both gari and eba, bulk density of gari, swelling index of gari, and textural properties of eba such as hardness and gumminess. The specific methodologies and measurement units for each trait are detailed in Table 1.

**TABLE 1:** Trait, class, and phenotyping method of the 12 traits assessed in the present study.

Traits	Class	Phenotyping method
Gari (%)	Processing traits	Gari (%) was calculated as a percentage of the initial weight of starting roots (Aghogho et al., 2022).
Peel loss (%)	Processing traits	Peel loss (%) was calculated as a percentage of the initial weight of starting roots (Aghogho et al., 2022).
L* gari	Color parameters	A chroma meter (CR‐400 Konica Minolta) was used to measure the Commission on Illumination (CIE) tristimulus L*(white/black), a*(red/green), and b* (yellow/blue) parameters. Gari and eba samples for color measurement were prepared as described by Aghogho et al. (2023).
a* gari		
b* gari		
a* eba		
b* eba		
L* eba		
Bulk density (g cm^−3^)	Functional properties	A 250‐mL graduated measuring cylinder was filled with gari samples and gently tapped on the laboratory bench about 50 times until they were leveled to the 250 mL mark (Aghogho et al., 2023).
Swelling index (SI)		10 g of gari samples was placed in a 100‐mL measuring cylinder and the initial volume was measured. Cold distilled water was added to the 100 mL mark of the cylinder and allowed to sit for 4 h before observing the final volume of gari. SI was calculated to be a multiple of the original volume (Aghogho et al., 2023).
Eba hardness (g)	Textural properties	Eba hardness and gumminess were measured using the textural profile analyzer (TA‐XTplus stable Microsystem) following the standard operating procedure of RTBFoods (https://doi.org/10.18167/agritrop/00604).
Eba gumminess (%)		

### 2.5 Diversity Arrays Technology sequencing (DArTseq) genotyping and genotypes filtering

Leaf samples were collected in 96‐well boxes and freeze‐dried to preserve DNA integrity. DNA extraction, genotyping, and SNP calling for each sample were performed using the DArTseq genotyping platform (https://www.diversityarrays.com/technology‐and‐resources/dartreseq/). Twelve of the 200 genotypes with more than 20% of their SNP marker data missing were excluded from the association analysis. SNPs with a missing call rate above 50% and a minor allele frequency (MAF) below 5% in the remaining genotypes were also removed. This resulted in a final dataset of 53,150 markers, which were used for GWAS, population structure, and genetic kinship analyses. SNP marker quality control was conducted using the Trait Analysis by Association, Evolution, and Linkage (TASSEL) software (Bradbury et al., 2007).

### 2.6 Statistical analysis

#### 2.6.1 Phenotype data analysis

Summary statistics for phenotypic data were done using the function stat.desc from the pastesc package and visualized using violin‐imposed boxplot in the R software.

A linear mixed model was used to obtain the best linear unbiased predictions (BLUPs) for each genotype. The lme4 package in the R software was used to fit the model (Bates, 2007) following the below statistical representation:

$$\begin{array}{ccc} y_{ijkl} & = & \mu +\mathrm{chec}k_{i}+\;env_{j}+\mathrm{Bloc}k_{k}\left(\mathrm{en}v_{i}\right)+\mathrm{ge}n_{k}\\ & & +\;\mathrm{en}v_{i}\times \mathrm{ge}n_{i}+\varepsilon_{ijkl} \end{array}$$

with $u∼N(0,I\sigma_{u}^{2})$ and $e∼N(0,I\sigma_{e}^{2})$, where **y** is the response vector of a trait for a given location, *μ* is the grand mean, $\mathrm{chec}k_{i}$ are the checks present in each block, $\mathrm{en}v_{i}\;$ is the environment effect, $\mathrm{Bloc}k_{k}\left(\mathrm{en}v_{i}\right)$ is the block within environment effect, $\mathrm{ge}n_{k}$ is the genotypic effect, $\mathrm{en}v_{i}\times gen_{i}$ is the genotype by environment effect, and $\varepsilon_{ijkl}\;$ is the residual. Variance was partitioned and used to estimate broad sense and SNP‐based heritability.

Association and correlation analysis was performed using the obtained deregressed BLUPs values as phenotypes. Deregressed BLUPs were estimated from the BLUPs obtained from the above model using the formula suggested by Garrick et al. (2009) and given as follows:

$$\mathrm{Deregressed}\;\mathrm{BLUP}\;=\frac{\mathrm{BLUPs}}{\left(1-\frac{\mathrm{PEV}}{\sigma_{g}^{2}}\right)}$$

where PEV is the predicted error variance of the BLUP and $\sigma_{g}^{2\;}$ is the genotypic variance component. Correlation analysis was carried out using the cor package and visualized using ggcorrplot in core R version 4.1.1 (R Core Team, 2020)

#### 2.6.2 Estimation of Genetic Parameters

##### Heritability estimates

Broad‐sense heritability was estimated as suggested by Piepho and Möhring (2007) and given as follows:

$$\;H^{2}=\sigma_{g}^{2}/\left(\sigma_{g}^{2}\;+\left(\sigma_{ge}^{2}/e\right)+\left(\sigma_{e}^{2}/er\right)\right)$$

where $H^{2}$ is the broad‐sense heritability estimate across all environments, $\sigma_{g}^{2}$ is genotypic variance, $\sigma_{ge}^{2}$ is the variance of genotype by environment interaction, $\sigma_{e}^{2}$ is the residual variance, *e* is the number of environments, and *r* is the number of replications.

SNP‐based heritability was estimated as suggested by Yang et al. (2017) and given as follows:

$$h_{\mathrm{SNP}}^{2}=\sigma_{g\left(\mathrm{SNP}\right)}^{2}\;/\left(\sigma_{g\left(\mathrm{SNP}\right)}^{2}+\sigma_{e}^{2}\right)$$

where $\sigma_{g(\mathrm{SNP})}^{2}$ is the SNP‐derived genetic variance and $\sigma_{e}^{2}$ is the SNP‐derived error variance.

SNP‐based genetic variance and SNP‐derived error variance were extracted using a compressed linear mixed model in TASSEL.

##### Genetic and phenotypic variation estimates

The phenotypic and genotypic coefficients of variation as well as genetic advance (GA) and genetic advance as percentage of mean (GAM) were estimated as per the formula prescribed by Burton and Devane (1953).

Phenotypic coefficient of variance

$$\mathrm{PCV}=\frac{{\left[\sigma_{p}^{2}\right]}^{1/2}}{\mu }\times 100$$

Genotypic coefficient of variance

$$\mathrm{GCV}\;=\frac{{\left[\sigma_{g}^{2}\right]}^{1/2}}{\mu }\;\times 100$$

Genetic advance

$$\mathrm{GA}=\left(K\right)\;{\left[\sigma_{p}^{2}\times \left(h_{\mathrm{SNP}}^{2}\right)\right]}^{1/2}$$

Genetic advance as a percentage of mean

$$\mathrm{GA}\%=\frac{\mathrm{GA}}{\mu }\;\;\times 100$$

where $\sigma_{p}^{2}$ is the phenotypic variance, $\sigma_{g}^{2}$ is the genotypic variance, μ is the mean, GA is the expected GA, *K* is the standardized selection differential at 5% selection intensity (*K* = 2.063), ${\left[\sigma_{p}^{2}\right]}^{1/2}$ is the phenotypic standard deviation on a mean basis, and $h_{\mathrm{SNP}}^{2}$ is the SNP‐based heritability.

### 2.7 Population structure analysis

Linkage disequilibrium (LD) pruning was conducted using Plink 1.9 to select 16,210 SNP markers with a pairwise LD value of <0.5 within each 50‐SNP window size. These LD‐pruned SNPs were subsequently used for population structure analysis, including hierarchical clustering and ADMIXTURE analysis. ADMIXTURE uses a model‐based clustering algorithm to assign proportions of ancestry to each individual based on their genotype data. For this study, a range of subpopulation counts (*K*) from 2 to 15 was tested to identify distinct ancestries. A 10‐fold cross‐validation procedure was employed to ensure the accuracy of the ADMIXTURE analysis results.

### 2.8 Genome‐wide association analyses

For each trait, GWAS was conducted using both single‐locus and multi‐locus models to identify significant SNP associations. The single‐locus model utilized was the compressed mixed linear model (CMLM), as recommended by Zhang et al. (2010). This model helps mitigate false positives by incorporating population structure (**Q**) and kinship (**K**) matrices into its corrections. Conversely, the multi‐locus model employed was the Bayesian information and linkage disequilibrium iteratively nested keyway (BLINK), as suggested by Huang et al. (2019), which addresses both false positives and false negatives by incorporating pseudo‐QTN as covariates. Both models were implemented using the Genome Association and Prediction Integrated Tool version 3 R package (J. Wang & Zhang, 2021). False discovery rate and marker allele frequency thresholds were set at 0.05.

Significant SNPs were initially identified using the Bonferroni correction *p*‐value threshold of −log10(*p*) = 6.00. However, for traits where this threshold proved too stringent, a more relaxed threshold of −log10(*p*) = 4.00 was applied based on the insights from quantile–quantile (QQ) plots and the distribution of *p*‐values, as discussed in the literature (Ji et al., 2021; Kristensen et al., 2019; Lou et al., 2021).

Manhattan plots of top SNPs from the CMLM and BLINK results were visualized using the ggplot2 package in R (Wickham, 2016). QQ plots were used to visualize the results of GWAS using the CMplot package in R software (Yin, 2020).

### 2.9 Linear regression and dependence of peak SNPs

We assessed the linear dependence of peak SNPs by fitting a linear regression model with peak SNPs at the identified loci as independent variables and the traits' BLUPS as the response variables. We used the *olsrr* package (Hebbali & Hebbali, 2017) to perform collinearity diagnostics on the output from the linear regression model. SNPs with variance inflation factors (VIFs) above 50 and infinity (inf) were dropped until only SNPs with VIF <5 were left in the model. The *R* ^2^ and non‐collinear peak SNPs were visualized using a bar plot from ggplot2.

### 2.10 Candidate gene identification

Candidate gene identification analysis was performed using non‐correlated significant SNPs selected from the GWAS results. The significant SNPs were mapped to their corresponding genes or to surrounding genes using the genome annotation available as a general feature format (gff3) file, M. esculenta_305_v6.1.gene.gff3, of the cassava reference genome v6 .1 available in Phytozome v12.1 (https://phytozome‐next.jgi.doe.gov/). The gff3 file was converted to a browser extensible data (bed) file using the gff2bed function from the bedops program (Neph et al., 2012). The closestBed function from bedtools (Quinlan & Hall, 2010) was then used to identify genes in the LD decay regions and then narrowed down to genes closest to significant SNPs. Gene ontology annotation was carried out on the plant Ensembl website (https://plants.ensembl.org/index.html). The National Center for Biotechnology Information genome data viewer using Manihot esculenta v6.1 (GCF_001659605.1) was used to confirm genes not found in the Ensembl plant.

## 3 RESULTS

### 3.1 Phenotypic variation and relationships among yield and quality of gari‐related traits

**FIGURE 1:**
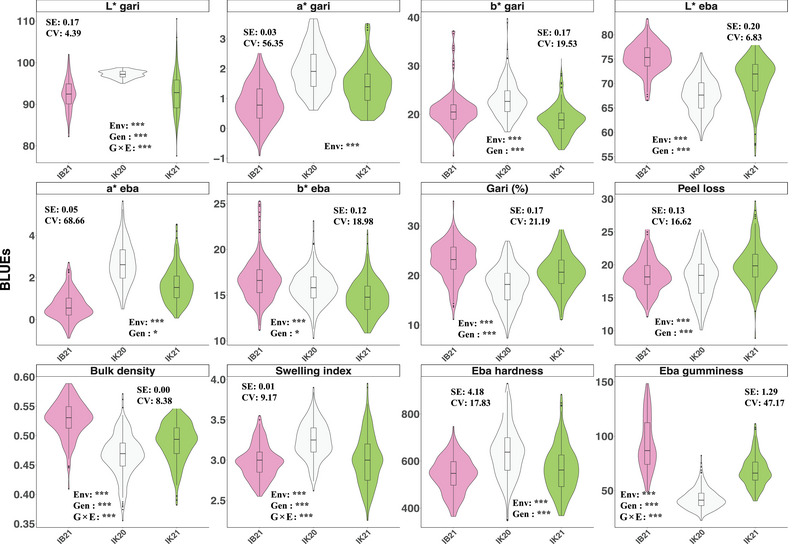
Summary statistics for yield and quality of gari‐related traits. CV, coefficient of variation; SE, standard error; BLUEs, best linear unbiased estimates.

Comprehensive statistical information on 12 essential traits related to gari yield and the quality of gari and eba are reported in Figure 1 and Table S2. The data showed significant genetic and environmental variation for all traits except for a* gari. Genotype by environment interaction was only significant in a couple of traits (L* gari, bulk density, swelling index, and eba gumminess). Across all measured traits, phenotypic variations exceeding two‐fold differences between maximum and minimum values were observed, with an average coefficient of variation (CV) of 25%. The color parameter for eba (a*) exhibited a significantly higher CV (67%). These differences observed between the maximum and minimum values are an indication of broad phenotypic variability within the association panel.

### 3.2 Estimates of variance components for yield and quality of gari‐related traits

To assess the impact of additive and nonadditive genetic effects on the observed phenotypic values, we computed estimates for broad‐sense and genomic heritability estimates from variance components. Estimates of these variance components for each of the studied traits are presented in Table 2. SNP‐based heritability ranged from 21% (peel loss) to 70% (b* gari and b* eba). Traits such as a* eba, b* eba, L* eba, swelling index, eba hardness, and eba gumminess exhibited higher SNP‐heritability estimates (>0.5). Phenotype‐based broad‐sense heritability ranged from 6% (L* gari) to 63% (b* gari and peel loss). Traits such as gari yield (%), peel loss, L* gari, a* gari, b* gari, and bulk density showed lower heritability estimates (<0.4). We observed that SNP‐based heritability was generally higher than phenotype‐based broad‐sense heritability. The phenotypic coefficient of variation (PCV) for all traits was relatively high, ranging from 4.23% to 58.91%, indicating substantial variation and, consequently, potential for improvement through selection. The genetic coefficient of variation (GCV) values were moderate, ranging from 0.52% to 16.86%. These results suggest considerable potential for phenotypic improvement across all traits through targeted selection.

**TABLE 2:** Estimates of variance components for gari yield and quality‐related traits.

Traits	*H* ^2^	$h_{\mathrm{SNP}}^{2}$	PCV (%)	GCV (%)	GA	GAM
Gari (%)	0.62	0.37	17.50	11.04	2.71	13.37
Peel loss (%)	0.63	0.21	11.27	9.51	0.92	4.89
L* gari	0.06	0.35	4.23	0.52	2.89	3.06
a* gari	0.15	0.27	41.60	13.05	0.34	23.04
b* gari	0.63	0.70	16.19	10.41	4.95	23.38
a* eba	0.23	0.41	58.91	16.86	0.90	50.01
b* eba	0.36	0.70	10.64	7.25	2.44	15.36
L* eba	0.35	0.40	5.91	2.01	3.46	4.88
Eba hardness	0.30	0.58	13.06	5.87	91.83	15.62
Eba gumminess	0.13	0.47	53.29	6.97	34.91	51.67
Swelling index	0.34	0.45	8.35	3.16	0.25	7.95
Bulk density	0.62	0.39	8.79	3.98	0.04	7.34

Abbreviations: GA, genetic advance, GAM, genetic advance as percentage of mean; GCV, genotypic coefficient of variation; *H* ^2^, broad sense heritability; $h_{\mathrm{SNP}}^{2}$, SNPs‐based heritability; PCV, phenotypic coefficient of variation.

### 3.3 Correlation analysis for yield and quality of gari and eba‐related traits

**FIGURE 2:**
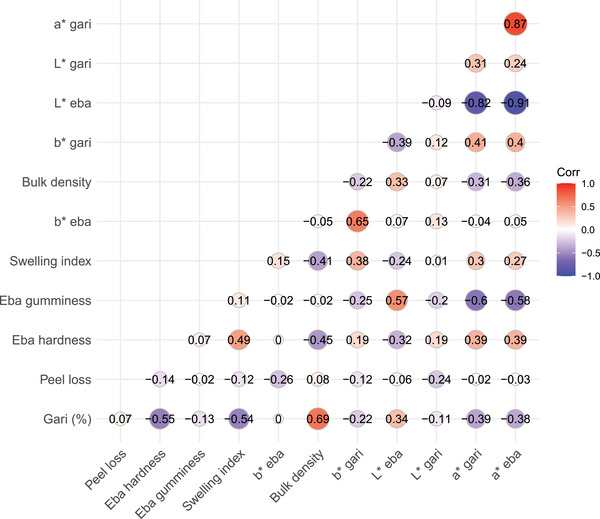
Correlation coefficient using deregressed best linear unbiased predictions (BLUPs) for 12 traits.

Significant variations were observed in the phenotypic correlations for gari yield, ranging from a negative correlation of −0.55 with eba hardness and swelling index to a positive correlation of 0.64 with bulk density (Figure 2). Notably, peel loss exhibited low, negative correlations with gari L* (−0.24, *p* < 0.001) and b* eba (−0.26, *p* < 0.001). Eba hardness correlated positively with eba a* (0.27, *p* < 0.01), a* gari (0.27, *p* < 0.01), and the swelling index (0.47, *p* < 0.01), and negatively with bulk density (−0.39, *p* < 0.01).

Eba gumminess showed moderate, negative correlations with a* eba (−0.55, *p* < 0.01) and a* gari (−0.54, *p* < 0.01), and a positive correlation with L* eba (0.56, *p* < 0.01). The swelling index was positively correlated with gari a* (0.34, *p* < 0.01) and eba a* (0.27, *p* < 0.01), and negatively with bulk density (−0.36, *p* < 0.01).

For color parameters, b* gari and b* eba displayed a high positive correlation (0.81, *p* < 0.001). Eba a* was significantly correlated with Leba (−0.87, *p* < 0.05), L gari (0.25, *p* < 0.05), and a* gari (0.81, *p* < 0.001). Additionally, a* gari was significantly negatively correlated with L* eba (−0.74, *p* < 0.001) and positively with L* gari (0.31, *p* < 0.01).

### 3.4 Genotypic relationships and informativeness in SNP data

**FIGURE 3:**
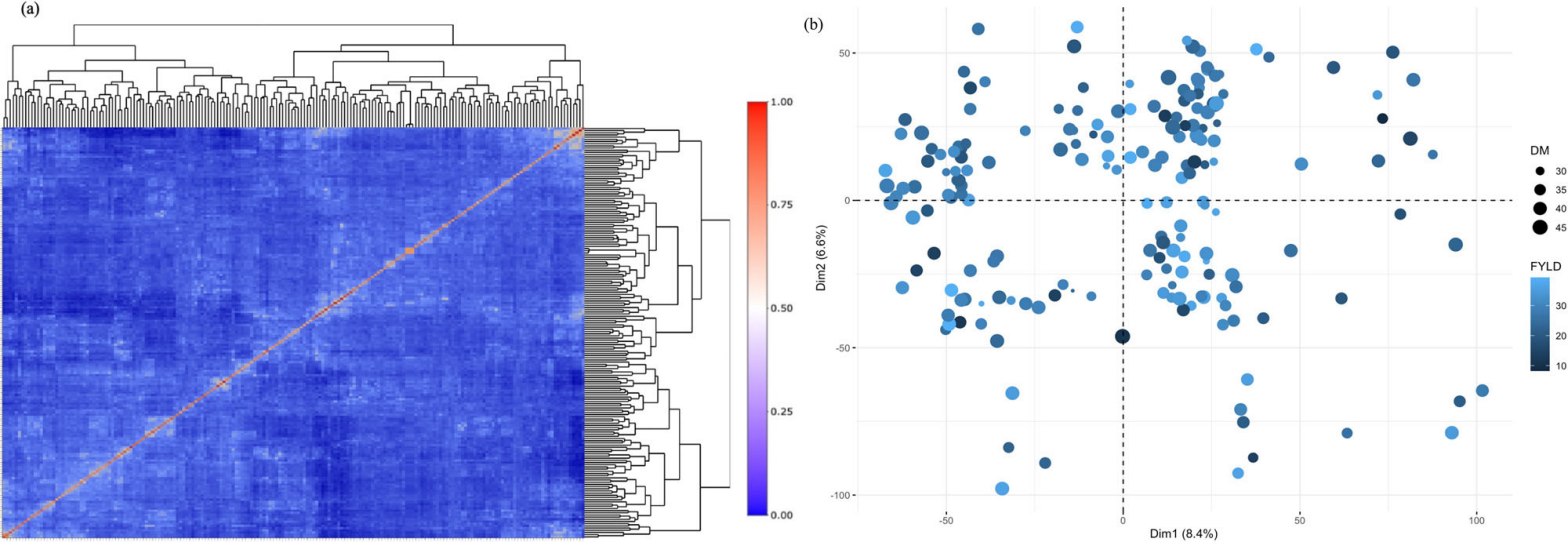
(a) Heat map of single nucleotide polymorphism (SNP)‐based relationship matrices for 200 cassava genotypes, showing genotype relatedness; (b) Scatter plot of first and second principal component of 200 genotypes showing marker informativeness across genotypes. DM, dry matter content; FYLD, fresh root yield (t/ha).

Cluster analysis using kinship matrix (Figure 3a) as well as principal component analysis (Figure 3b) was used to assess the genetic relationship among the accessions in the GWAS panel. PC1 and 2 explained about 15% of the total genetic variation, suggesting broad genetic diversity. In terms of their phenotypic characteristics, the genotypes exhibited a variation in root dry matter content and fresh root weight, ranging from 21.02% to 44.8% and 10 to 38 kg, respectively.

### 3.5 Distribution of SNPs, LD and population structure

**FIGURE 4:**
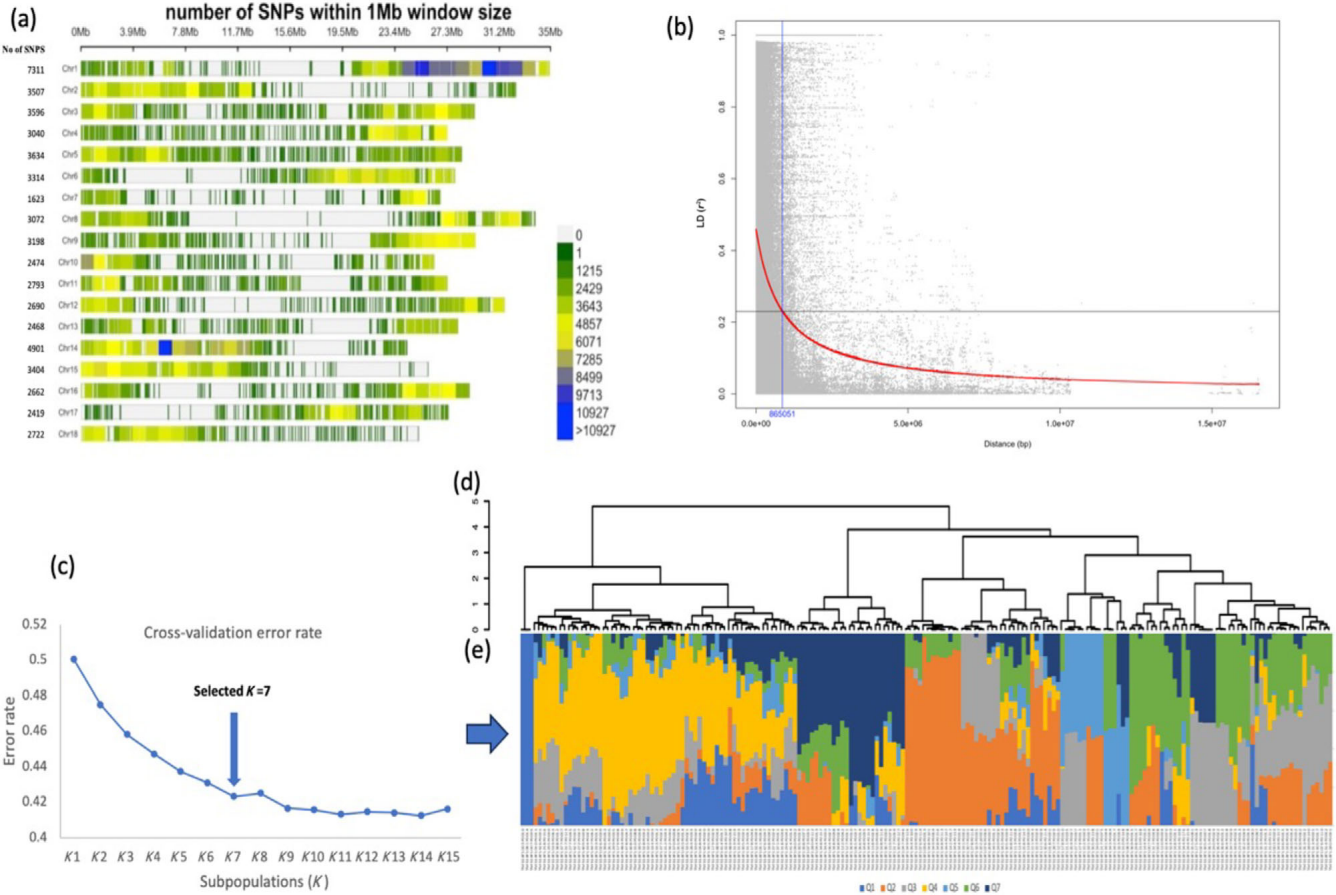
(a) Genome‐wide single nucleotide polymorphism (SNP) distributions on 18 chromosomes of cassava. The horizontal axis displays the chromosome length in base pairs; the vertical axis on the left displays the number of SNPs; 0–10,927 color‐coded legend insert indicates the density of SNPs; the number of SNPs is also indicated for each of the 18 chromosomes. (b) Linkage disequilibrium (LD, *r* ^2^) decay plot of 53,150 marker pairs as a function of physical distance (bp) for the 188‐cassava genotype. (c) The population structure of cassava accessions for 188 genotypes based on 16,210 SNPs and a 10 fold cross‐validation error rate for *K* = 2 to *K* = 15. (d) Hierarchical clustering (Ward's minimum variance method) dendrogram. (e) Individual ancestry estimated ADMIXTURE analysis (*K* = 7). The colored sections within each bar indicate membership of the genotypes in the different subpopulations.

Genome‐wide SNP distributions on 18 chromosomes of cassava are shown in Figure 4a. SNPs coverage per chromosome varied from 1623 on chromosome 7 to 7311 on chromosome 1, indicating considerable differences in SNP density across chromosomes. Figure 4b shows the decay of genome‐wide LD for 188 cassava genotypes. The LD decay across the whole genome had a maximum LD (*r* ^2^) value of 0. 46 and decreased to 0.23 at 10 kb, suggesting rapid genetic recombination and potential for higher resolution mapping (Figure 4b). The population structure of the 188 cassava genotypes based on 16,210 SNPs is presented in Figure 4e. The population stratification inferred by using the model‐based ADMIXTURE clustering method indicated the presence of seven subpopulations (Figure 4e).

### 3.6 Genome‐wide association results

**FIGURE 5:**
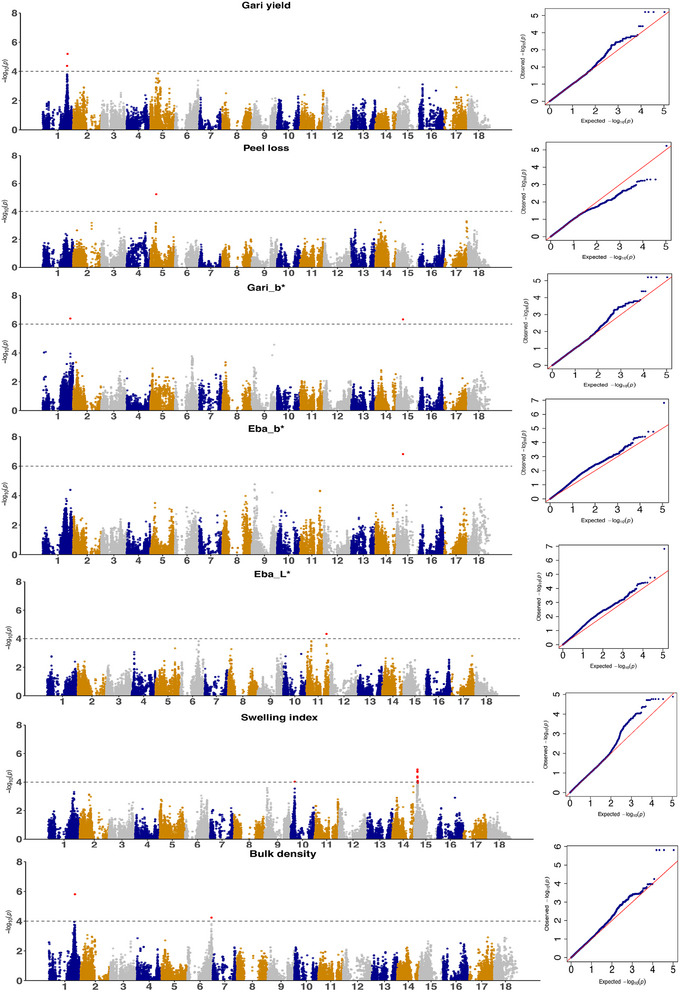
Significant single nucleotide polymorphisms (SNPs) associated with gari yield and quality of gari‐related traits identified using single and multi‐locus genome‐wide association study (GWAS) methods.

Only traits with broad‐sense heritability estimates above 0.30 were analyzed in the GWAS (Table 2). This resulted in eight traits related to gari yield and quality of gari and eba being considered for association analysis: L* eba, b* eba, bulk density, b* gari, eba hardness, peel loss, and gari yield (%). A combined total of 58 unique genomic regions associated with these traits were identified using CMLM and BLINK models, with nine and eight unique regions found exclusively by each model, respectively, and 41 regions identified by both (Figure 5). The most significant trait‐marker associations, including specific genomic regions, minor allele frequencies, *p*‐values, and the proportion of phenotypic variance explained by the SNPs, are provided in Table S3. In the following sections, we present the results for each trait.

The GWAS to gari productivity traits revealed several significant SNP associations across multiple chromosomes. For gari yield (%), seven significant SNPs on chromosome 1 were associated with variation in gari yield (%) (Figure 5). The most significant loci were all on chromosome 1 and were tagged by markers S1_28607143 (*p*‐value = 6.63E‐06), S1_28607159 (*p*‐value 6.63E‐06), S1_28607178 (*p*‐value = 6.63E‐06), and S1_28607187 (*p*‐value = 6.63E‐06). These four markers had an MAF of 0.34 (Table S3). A single genomic region on chromosome 5 was found to be significantly associated with peel loss and tagged by S5_6824266 (*p*‐value = 4.99E‐06) with an MAF of 0.07 and explaining 11.83% of the phenotypic variance.

The GWAS for color‐related traits in gari and eba revealed several significant associations across multiple chromosomes. For b* gari, which measures the yellowness of gari, eight loci were identified on chromosomes 1, 11, and 15. The locus on chromosome 11, tagged by S11_23058961 (*p*‐value of 4.65E‐09) with an MAF of 0.47, explained 8.76% of phenotypic variance. In contrast, L* eba, assessing the brightness of eba, was linked to a significant locus on chromosome 11 at S11_23133377 (*p*‐value = 3.21E‐05), which had an MAF of 0.19 and accounted for 8.0% of the variance. Additionally, the yellowness of eba, denoted as b* eba, was associated with a locus on chromosome 15. The marker, S15_7457511 (*p*‐value = 1.53E‐079), with an MAF of 0.470, had a %PVE of 8.85%.

Several loci were found to be associated with gari and eba physicochemical traits. For the swelling index, a trait indicative of gari's ability to absorb water and swell, we found significant association signals on chromosomes 10 and 15. The most significant locus on chromosome 15 was tagged by marker S15_3653278 (*p*‐value = 1.13E‐05), which has an MAF of 0.44. The bulk density of gari, which relates to its compactness and heaviness, was associated with five SNP markers on chromosomes 1 and 6. Four of these markers were located on chromosome 1, with the most prominent being S1_28607143, characterized by a *p*‐value of 2.70E‐11, with an MAF of 0.34 and a %PVE of 12.1%. Conversely, eba hardness, which affects the firmness and mouthfeel of the prepared food product, did not reveal any significant associations with the markers analyzed in this study. The absence of notable loci implies that eba hardness may be controlled by complex genetic factors that are not easily captured by the current SNP array or may be more influenced by environmental factors and postharvest processing methods.

### 3.7 Linear regression and dependence of peak SNPs

**FIGURE 6:**
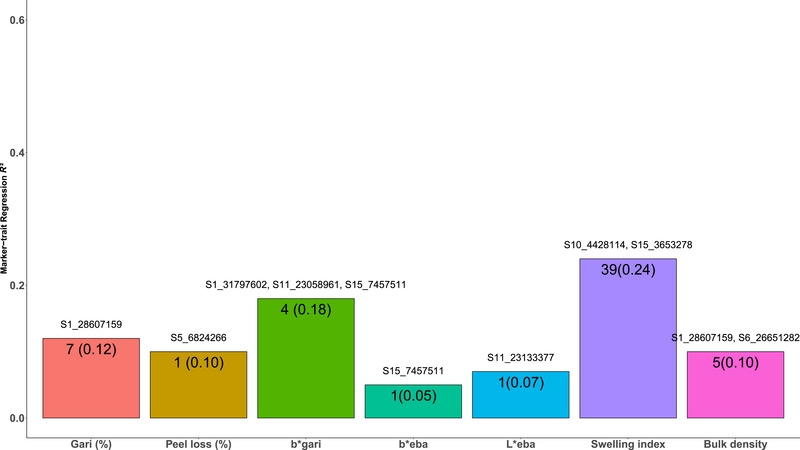
Multiple marker‐trait regression barplot across traits and non‐collinearity peak single nucleotide polymorphisms (SNPs). Numbers in the barplot denote the number of loci in the regression model with the *R* ^2^ in the bracket.

To assess the strength of the association of Peak SNPs with studied traits, we performed a multiple linear model using peak SNPs at the identified loci (Figure 5) as independent variables against the traits BLUPs as the response variables. The *R* ^2^ values for the peak SNPs at the identified loci ranged from 0.05% (b* eba) to 0.24% (swelling index) (Figure 6). The markers for L* eba, bulk density, and peel loss had the lowest predictive ability (*R* ^2^ < 0.1). The result of the multicollinearity analysis revealed that among the 39 peak SNPs observed for swelling index, two were found to be non‐correlated (Figure 6; Table S4). Among the five peak SNPs associated with bulk density, two were found to be uncorrelated.

### 3.8 Putative candidate gene identification

Non‐correlated significant SNPs were further examined to identify annotated genes with putative functions using the M. esculenta 305_v6.1.gene.gff3 file of the cassava reference genome available on Phytozome v12.1. The number of genes identified using the LD decay regions varies across traits, ranging from 117 for the b*gari trait to 268 for traits such as gari percentage and bulk density of gari (Table S5). Further, we then narrowed down the search to genes close to the significant SNPs (Table 3).

**TABLE 3:** Putative candidate genes‐closest to significant single nucleotide polymorphisms (SNPs) associated with gari yield and quality.

Traits	Significant SNP position	Closest gene chr	Closest gene start	Closest gene end	Closest gene ID	Coding protein
Gari (%)	S1_28607159	1	28604014	28607796	Manes.01G189200	Ubiquitin‐conjugating enzyme 2
peel_loss	S5_6824266	5	6822830	6823115	Manes.05G086700	Hypothetical protein
b_gari	S1_31797602	1	31797590	31800894	Manes.01G236900	Cysteine proteinases superfamily protein
b_gari	S11_23058961	11	23058367	23061964	Manes.11G121400	Leucine‐rich repeat protein kinase family protein
b_gari	S15_7457511	15	7453476	7478527	Manes.15G100800	hypothetical protein
b_eba	S15_7457511	15	7453476	7478527	Manes.15G100800	hypothetical protein
L_eba	S11_23133377	11	23132908	23135282	Manes.11G122000	B‐box type zinc finger
Swelling index	S10_4428114	10	4427087	4440188	Manes.10G046600	Phosphoglucosamine mutase family protein
Swelling index	S15_3653278	15	3649673	3653914	Manes.15G048700	Pectin lyase‐like superfamily protein
Bulk density	S1_28607159	1	28604014	28607796	Manes.01G189200	Ubiquitin‐conjugating enzyme 2
Bulk density	S6_26651282	6	26649169	26651496	Manes.06G164300	Ribosomal protein L14p/L23e family protein

The candidate gene Manes.01G189200, coding for ubiquitin‐conjugating enzyme E2 protein, was found closest to the significant SNP (SI_28607159) identified for gari yield (%) and bulk density of gari. An uncharacterized protein was found to be closest to the significant SNP (S5_6824266) identified for peel loss. Gene identification for color‐related traits for gari and eba revealed that candidate gene Manes.11G121400 encodes a leucine‐rich repeat (LRR) protein kinase family protein and was found as the closest gene to S11_23058961. For eba brightness (L* eba) and the candidate gene Manes.11G122000, encoding for the B‐box (BBX) zinc finger family protein was found closest to S11_23133377.

Two putative candidate genes were found to be closest to S10_4428114 and S15_3653278 SNPs identified to be associated with swelling encoding for proteins belonging to the phosphoglucosamine mutase (PGM) family (Manes.10G046600) and pectin lyase‐like (PEL) superfamily (Manes.15G048700), respectively.

## 4 DISCUSSION

The genetic architecture of gari yield and quality is essential for varietal development and acceptability. Despite advances in studying cassava root quality traits, the genetic blueprint for its processed products remains unexplored. This study aimed to estimate the GA and identify genomic regions and candidate genes associated with cassava gari yield (%) and quality. While significant phenotypic variation was observed for all traits, L* gari and a* gari showed minimal variation. This is because L* and a* measurements capture the red‐green (a*) and lightness‐darkness (L*) spectrums of color. However, these spectrums alone might not fully represent the complexities of color variation observed in gari. This was evident by the more than two‐fold difference between the minimum and maximum values, and the significant genetic and environmental variation. The range and mean values observed for the traits under study were consistent with what was reported for gari produced from frozen roots (Oyeyinka et al., 2019). The similarity in results may be due to the same range of genetic diversity in the genetic materials or phenotyping methods used in both studies. However, the significant genetic variance offers a valuable resource for genetic improvement for these traits. The estimation of SNP‐based heritability is a valuable tool for quantifying the contribution of additive genetic variation to phenotypic variance. This is because it considers individual genetic variants' contribution to trait expression (Yang et al., 2017). We observed a higher SNP‐based than broad‐sense heritability for all traits measured except for gari yield, peel loss, and bulk density. This difference may be due to the influence of environmental and genotype by environment factors on phenotypic broad‐sense heritability (Nyquist & Baker, 1991) or other factors such as population stratification, LD, sample size, and genotyping data quality on SNP‐based heritability (Zhu & Zhou, 2020). Both phenotypic broad‐sense and SNP‐based heritability estimates were moderate to high, indicating the significant influence of additive genetics on these traits. This suggests promising prospects for genetic improvement and highlights the repeatability and reproducibility of the experimental procedures. All traits exhibited PCV greater than GCV, indicating significant influence of genetics and environment on traits. This trend is consistent with other studies involving cassava yield traits such as dry matter content and starch content (Peprah et al., 2020). The GAM values obtained in this study are within the range of values reported for other cassava studies (Adjebeng‐Danquah et al., 2017; Akinwale et al., 2010). Despite the lower SNP‐based heritability estimate of eba gumminess (0.47) compared to b* eba (0.70), we observed similar GAM. This suggests that while eba gumminess likely has higher nonadditive variance and environmental influence, genetic progress remains achievable for both traits in each selection cycle (Pradeepkumar et al., 2001). Traits with high estimates of genetic improvement potential (PCV, GCV, GA, and GAM) offer opportunities for genetic improvement. This study revealed moderate values for these measures, suggesting potential for genetic improvement in gari yield and quality traits.

Trait correlation plays a pivotal role in improving the accuracy of selection for complex traits via indirect selection. However, negative correlations between two desirable traits could potentially hinder simultaneous genetic progress. Significant correlations among traits may arise from genetic linkage and/or pleiotropic effects of different genes. This study revealed a stronger positive correlation between eba hardness and both bulk density and swelling index compared to what was reported by Awoyale et al. (2022). The difference could be attributed to variations in population size and location between the two studies. The findings from our study were consistent with the findings of Almazan (1992) for the negative correlation between swelling index and gari yield (%). The negative correlation between eba hardness and bulk density presents a significant challenge for breeders aiming to simultaneously improve both traits. Advanced breeding methods such as marker‐assisted selection and genomic selection will be necessary to identify genotypes with favorable combinations of eba hardness and bulk density. Also, multi‐trait selection strategies can aid breeders in navigating this negative correlation and achieving simultaneous improvement in both traits (Gamal El‐Dien et al., 2015; Merrick & Carter, 2021).

For a GWAS analysis, understanding the relationship among genotypes and assessing the informativeness of SNPs are crucial steps for identifying genetic variants associated with phenotypic traits. Previous studies have identified a correlation between root dry matter content and the yield and quality of gari (Almazan, 1992; Laya et al., 2018). Therefore, a population exhibiting the range of dry matter content reported in this study offer a platform for conducting gene discovery analysis. Also, the DArTseq genotyping platform generates a moderate to high density of markers, making it suitable for GWAS in cassava. The availability of numerous markers in the platform allows for the detection of genetic variations associated with complex traits. Compared to other genotyping platforms such as SNP arrays and whole‐genome sequencing, DArTseq offers a good balance between marker selection, cost, and marker density, making it more feasible for large‐scale association studies in cassava (Davey et al., 2011). DarTseq in association analysis for cassava has been reported for several marker discoveries studies in traits such as root tuber quality traits (Rabbi et al., 2017), flowering‐related traits (Baguma et al., 2024), and nitrogen use efficiency in cassava (Mbe et al., 2024).

Population structure and cryptic genetic relatedness can lead to misleading associations in GWAS, even with advanced statistical methods (Hellwege et al., 2017). To ensure accurate analysis, this study adopted a combination of methods to assess population structure and minimize spurious associations. This included the use of a 15‐fold cross‐validation ratio, estimating admixture ancestry, and performing hierarchical clustering. The analysis revealed 188 genotypes clustered into seven distinct subpopulations. Despite this structure, genotypes with low to high dry matter content were distributed across all subpopulations, suggesting that the population is suitable for association analysis.

GWAS has identified genes associated with traits like yield, disease resistance, and starch content in cassava (Zargar et al., 2015) and quality traits in cooked rice (Misra et al., 2018). Taking examples from previous GWAS works, this study focuses on putative candidate genes for product quality traits in cassava. The ubiquitin‐conjugating enzymes (E2s) found closest to significant SNPs related to gari (%) and bulk density are deeply involved in hormone signaling transduction (Santner & Estelle, 2010) and responses to biotic and abiotic stresses (Becker et al., 1993; Lee & Kim, 2011). This suggests a potential link to stress resilience which could indirectly influence yield and bulk density. In cassava, specific subfamilies of the ubiquiting‐conjugating (UBC) superfamily (UBC35 subfamily) have been associated with root development (Salcedo et al., 2014), a major factor in determining gari yield (%) and bulk density. In Arabidopsis, the UBC34 subfamily regulates the SUC2 transporter, influencing sucrose transport and potentially carbohydrate metabolism (Liu et al., 2020). In yeast, the UBC8 subfamily is critical for the degradation of fructose 1,6‐bisphosphatase 1 (Fbp1), a key element in carbohydrate metabolism (Schüle et al., 2000; Z. Wang et al., 2022). Given the link between carbohydrate metabolism, sucrose transport, and the accumulation of dry matter in cassava roots (Beyene et al., 2020), the implications of this gene in gari production are even more significant. This aligns with previous research by Teeken et al. (2021), which suggested a potential correlation between dry matter content and gari yield (%).

The LRR protein kinase, associated with variations in b* gari as described by Jones and Jones (1997), encompasses receptor serine‐threonine kinases and polygalacturonase‐inhibiting proteins. Previous research has drawn a connection between LRR protein kinase family protein expression and flour color in wheat (Ji et al., 2021). In melon fruit, the LRR protein has shown substantial correlations with the orange gene (CmOr), regulating the accumulation of β‐carotene (Chayut et al., 2021). The interaction between the orange gene and the PSY1 gene in cassava has a role in carotenoid accumulation (Jaramillo et al., 2022), but the relationship between the LRR protein kinase, orange gene, and PSY1 gene in cassava's carotenoid regulation remains unexplained.

The BBX family protein, which was found close to the significant SNPs associated with the brightness of eba (L*eba), comprises zinc‐finger transcription factors containing one or two highly conserved BBX motifs at their N‐termini (Fang et al., 2019). BBX proteins are key players in plant developmental and growth processes like seedling light response, shade avoidance, flowering time regulation, stress resilience, fruit pigmentation, and carotenoid accumulation (Kim et al., 2019; Soitamo et al., 2008; Wu, 2011; Xiong et al., 2019). In tomatoes (*Solanum lycopersicum*) and cucumber (*Cucumis sativus*), it functions by binding to the promoter region of phytoene synthase 1 (PSY1), leading to elevated carotenoid accumulation within the chloroplast (Cao et al., 2023; Obel et al., 2022; Xiong et al., 2019). Further research is needed to understand the regulatory mechanisms of BBX and its interactions with pigment‐related pathways in cassava roots.

For the swelling index of gari, two putative candidate genes were identified near significant SNPs. These genes are potentially involved in cellulose synthesis and degradation of the pectate component of the plant cell wall. The PGM family protein plays a role in the accumulation of reserve starch and tuber growth by catalyzing the interconversion of glucose 6‐phosphate and glucose 1‐phosphate (Fettke et al., 2010; Tauberger et al., 2000). A lower PGM activity reduces starch reserve and tuber growth and increases the production of uridine diphosphate‐glucose, a precursor of cellulose synthesis (Swissa et al., 1980). Cellulose has been shown to affect water distribution and absorption in wheat dough (Hu et al., 2022; Jiang et al., 2020). The PEL superfamily protein is known to be involved in the degradation of the pectate component of the plant cell wall by depolymerizing enzymes that degrade the polygalacturonic acid (Henrissat et al., 1995). This degradation initiates cell separation and wall loosening during fruit ripening (Sun & van Nocker, 2010; Voragen et al., 2009). Cell wall separation and loosening reduce the adhesion between cells, a crucial factor affecting cell hydration properties (Auffret et al., 1994; Kunzek et al., 1999; Müller & Kunzek, 1998). Pectin has been linked to water absorption capacity in cassava‐based products (Awoyale et al., 2023). Although our study identified associations between the swelling index of gari and both PGM and PEL, we did not observe a clear interaction between these genes. This complexity suggests that water cassava granules water absorption likely involves an intricate interplay of genetic factors beyond individual genes.

This study offers a valuable starting point for improving the yield and quality of gari by cassava breeders. Future steps include investigating the specific function of these genes, validating their roles and developing molecular markers for breeding selection, to improve traits related to yield and quality of gari.

## 5 CONCLUSION

This study represents a pioneering effort in cassava research, as it is the first to conduct a comprehensive GWAS to identify the underlying genetic factors influencing gari yield and the quality of gari and eba. This powerful approach identified nine significant SNPs closely associated with variations in measured traits. These identified polymorphisms hold immense potential as molecular markers enabling breeders to effectively select for desired traits for increased genetic gain. Nonetheless, research and experimental validation will help elucidate these genes’ functional roles and their potential contributions to gari yield and quality, ultimately advancing our knowledge and practical application in cassava breeding programs.

## AUTHOR CONTRIBUTIONS

**Cynthia Idhigu Aghogho**: Conceptualization; data curation; formal analysis; visualization; writing—original draft. **Siraj Ismail Kayondo**: Conceptualization; formal analysis; methodology; validation; visualization; writing—review and editing. **Saviour J. Y. Eleblu**: Supervision; writing—review and editing. **Adenike Ige**: Formal analysis; methodology; visualization; writing—review and editing. **Isaac Asante**: Supervision; writing—review and editing. **Samuel K. Offei**: Supervision; writing—review and editing. **Elizabeth Parkes**: Conceptualization; supervision; writing—review and editing. **Edwige Gaby Nkouaya Mbanjo**: Formal analysis; supervision; writing—review and editing. **Trushar Shah**: Data curation; formal analysis; writing—review and editing. **Chiedozie Egesi**: Conceptualization; funding acquisition; supervision; writing—review and editing. **Peter Kulakow**: Conceptualization; funding acquisition; supervision. **Ismail Y. Rabbi**: Conceptualization; formal analysis; funding acquisition; supervision; writing—review and editing.

## CONFLICT OF INTEREST STATEMENT

The authors declare no conflicts of interest.

## Supporting information

Table S1: Pedigree information of the GWAS panel.

Table S2: Summary statistics of all the traits measured.

Table S3: Marker‐traits information

Table S4: Multicollinearity analysis of significant single nucleotide polymorphisms (SNPs).

Table S5: Genes identified within the LD decay regions.

## Data Availability

The datasets analyzed for the current study and original GWAS output files are available at the https://cassavabase.org/ftp/manuscripts/Aghogho_et_al_2023/.

## References

[tpg220469-bib-0001] Abass, A. B. , Dziedzoave, N. T. , Alenkhe, B. E. , & James, B. D. (2013). Quality management manual for the production of gari. International Institute of Tropical Agriculture.

[tpg220469-bib-0002] Adjebeng‐Danquah, J. , Manu‐Aduening, J. , Gracen, V. E. , Asante, I. K. , & Offei, S. K. (2017). AMMI stability analysis and estimation of genetic parameters for growth and yield components in cassava in the forest and guinea savannah ecologies of Ghana. International Journal of Agronomy, 2017, Article 8075846. 10.1155/2017/8075846

[tpg220469-bib-0003] Aghogho, C. I. , Eleblu, S. J. , Bakare, M. A. , Kayondo, S. I. , Asante, I. , Parkes, E. , Kulakow, P. , Offei, S. , & Rabbi, I. Y. (2022). Genetic variability and genotype by environment interaction of two major cassava processed products in multi‐environments. Frontiers in Plant Science, 13, 974795. 10.3389/fpls.2022.974795 mmkckfffd 36325542 PMC9618686

[tpg220469-bib-0097] Aghogho, C. I. , Kayondo, S. I. , Maziya‐Dixon, B. , Eleblu, S. J. , Asante, I. , Offei, S. K. , & Rabbi, I. Y. (2023). Exploring genetic variability, heritability, and trait correlations in gari and eba quality from diverse cassava varieties in Nigeria. Journal of the Science of Food and Agriculture, 10–12. 10.1002/jsfa.12889 37515474

[tpg220469-bib-0004] Akinwale, M. G. , Akinyele, B. O. , Dixon, A. G. O. , & Odiyi, A. C. (2010). Genetic variability among forty‐three cassava genotypes in three agro‐ecological zones of Nigeria. Journal of Plant Breeding and Crop Science, 2(5), 104–109.

[tpg220469-bib-0005] Almazan, A. M. (1992). Influence of cassava variety and storage on gari quality. Tropical Agriculture, 69(4), 386–390.

[tpg220469-bib-0006] Auffret, A. , Ralet, M. C. , Guillon, F. , Barry, J. L. , & Thibault, J. F. (1994). Effect of grinding and experimental conditions on the measurement of hydration properties of dietary fibres. LWT‐Food Science and Technology, 27(2), 166–172. 10.1006/fstl.1994.1033

[tpg220469-bib-0007] Awoyale, W. , Oyedele, H. A. , Adesokan, M. , Alamu, E. O. , & Maziya‐Dixon, B. (2022). Can improved cassava genotypes from the breeding program substitute the adopted variety for gari production? Biophysical and textural attributes approach. Frontiers in Sustainable Food Systems, 6(6). https://www.frontiersin.org/articles/10.3389/fsufs.2022.984687/full

[tpg220469-bib-0099] Awoyale, W. , Olatoye, K. K. , & Maziya‐Dixon, B. (2023). Cassava pectin and textural attributes of cooked gari (*eba*) and fufu dough. In M. Ahmed (Ed.), Utilization of pectin in the food and drug industries. IntechOpen. https://www.intechopen.com/chapters/85583

[tpg220469-bib-0008] Baguma, J. K. , Mukasa, S. B. , Nuwamanya, E. , Alicai, T. , Omongo, C. A. , Ochwo‐Ssemakula, M. , Ozimati, A. , Esuma, W. , Kanaabi, M. , & Wembabazi, E. (2024). Identification of genomic regions for traits associated with flowering in cassava (*Manihot esculenta* Crantz). Plants, 13(6), 796. 10.3390/plants13060796 38592820 PMC10974989

[tpg220469-bib-0009] Bassey, E. E. (2018). Evaluation of nine elite cassava (Manihot *e*sculenta Crantz) genotypes for tuber and gari yields and gari quality in four locations in Akwa Ibom State, Nigeria. American Research Journal of Agriculture, 4(1), 1–10.

[tpg220469-bib-0100] Bates, D. (2007). Linear mixed model implementation in lme4. University of Wisconsin.

[tpg220469-bib-0011] Bechoff, A. , Tomlins, K. , Fliedel, G. , Becerra Lopez‐lavalle, L. A. , Westby, A. , Hershey, C. , & Dufour, D. (2018). Cassava traits and end‐user preference: Relating traits to consumer liking, sensory perception, and genetics. Critical Reviews in Food Science and Nutrition, 58(4), 547–567. 10.1080/10408398.2016.1202888 27494196

[tpg220469-bib-0012] Becker, F. , Buschfeld, E. , Schell, J. , & Bachmair, A. (1993). Altered response to viral infection by tobacco plants perturbed in ubiquitin system. The Plant Journal, 3(6), 875–881. 10.1111/j.1365-313X.1993.00875.x

[tpg220469-bib-0013] Beyene, G. , Chauhan, R. D. , Gehan, J. , Siritunga, D. , & Taylor, N. (2020). Cassava shrunken‐2 homolog MeAPL3 determines storage root starch and dry matter content and modulates storage root postharvest physiological deterioration. Plant Molecular Biology, 109, 283–299. 32270429 10.1007/s11103-020-00995-z PMC9163024

[tpg220469-bib-0014] Bradbury, P. J. , Zhang, Z. , Kroon, D. E. , Casstevens, T. M. , Ramdoss, Y. , & Buckler, E. S. (2007). TASSEL: Software for association mapping of complex traits in diverse samples. Bioinformatics, 23(19), 2633–2635. 10.1093/bioinformatics/btm308 17586829

[tpg220469-bib-0015] Burns, A. , Gleadow, R. , Cliff, J. , Zacarias, A. , & Cavagnaro, T. (2010). Cassava: The drought, war and famine crop in a changing world. Sustainability, 2(11), 3572–3607. 10.3390/su2113572

[tpg220469-bib-0016] Burton, G. W. , & Devane, D. E. (1953). Estimating heritability in tall fescue (*Festuca arundinacea*) from replicated clonal material. Agronomy Journal, 45(10), 478–481. 10.2134/agronj1953.00021962004500100005x

[tpg220469-bib-0017] Cao, J. , Yuan, J. , Zhang, Y. , Chen, C. , Zhang, B. , Shi, X. , Niu, R. , & Lin, F. (2023). Multi‐layered roles of BBX proteins in plant growth and development. Stress Biology, 3(1), Article 1. 10.1007/s44154-022-00080-z 37676379 PMC10442040

[tpg220469-bib-0018] Ceballos, H. , Kawuki, R. S. , Gracen, V. E. , Yencho, G. C. , & Hershey, C. H. (2015). Conventional breeding, marker‐assisted selection, genomic selection and inbreeding in clonally propagated crops: A case study for cassava. Theoretical and Applied Genetics, 128, 1647–1667. 10.1007/s00122-015-2555-4 26093610 PMC4540783

[tpg220469-bib-0089] Chang , & Wu, S.‐H. (2011). COP1‐mediated degradation of BBX22/LZF1 optimizes seedling development in Arabidopsis. Plant Physiology, 156(1), 228–239. 21427283 10.1104/pp.111.175042 PMC3091042

[tpg220469-bib-0019] Chang, S. , Chen, Q. , Yang, T. , Li, B. , Xin, M. , Su, Z. , Du, J. , Guo, W. , Hu, Z. , Liu, J. , Peng, H. , Ni, Z. , Sun, Q. , & Yao, Y. (2022). Pinb‐D1p is an elite allele for improving end‐use quality in wheat (*Triticum aestivum* L.). Theoretical and Applied Genetics, 135(12), 4469–4481. 10.1007/s00122-022-04232-7 36175525 PMC9734229

[tpg220469-bib-0020] Chayut, N. , Yuan, H. , Saar, Y. , Zheng, Y. , Sun, T. , Zhou, X. , Hermanns, A. , Oren, E. , Faigenboim, A. , & Hui, M. (2021). Comparative transcriptome analyses shed light on carotenoid production and plastid development in melon fruit. Horticulture Research, 8, 112. 10.1038/s41438-021-00547-6 33931604 PMC8087762

[tpg220469-bib-0021] Davey, J. W. , Hohenlohe, P. A. , Etter, P. D. , Boone, J. Q. , Catchen, J. M. , & Blaxter, M. L. (2011). Genome‐wide genetic marker discovery and genotyping using next‐generation sequencing. Nature Reviews Genetics, 12(7), 499–510. 10.1038/nrg3012 21681211

[tpg220469-bib-0022] Ezenwaka, L. , Del Carpio, D. P. , Jannink, J.‐L. , Rabbi, I. , Danquah, E. , Asante, I. , Danquah, A. , Blay, E. , & Egesi, C. (2018). Genome‐wide association study of resistance to cassava green mite pest and related traits in cassava. Crop Science, 58(5), 1907–1918. 10.2135/cropsci2018.01.0024

[tpg220469-bib-0023] Fang, H. , Dong, Y. , Yue, X. , Hu, J. , Jiang, S. , Xu, H. , Wang, Y. , Su, M. , Zhang, J. , & Zhang, Z. (2019). The B‐box zinc finger protein MdBBX20 integrates anthocyanin accumulation in response to ultraviolet radiation and low temperature. Plant, Cell & Environment, 42(7), 2090–2104. 10.1111/pce.13552 30919454

[tpg220469-bib-0024] Fettke, J. , Albrecht, T. , Hejazi, M. , Mahlow, S. , Nakamura, Y. , & Steup, M. (2010). Glucose 1‐phosphate is efficiently taken up by potato (*Solanum tuberosum*) tuber parenchyma cells and converted to reserve starch granules. New Phytologist, 185(3), 663–675. 10.1111/j.1469-8137.2009.03126.x 20028468

[tpg220469-bib-0025] Gamal El‐Dien, O. , Ratcliffe, B. , Klápště, J. , Chen, C. , Porth, I. , & El‐Kassaby, Y. A. (2015). Prediction accuracies for growth and wood attributes of interior spruce in space using genotyping‐by‐sequencing. BMC Genomics, 16, Article 370. 10.1186/s12864-015-1597-y 25956247 PMC4424896

[tpg220469-bib-0026] Garrick, D. J. , Taylor, J. F. , & Fernando, R. L. (2009). Deregressing estimated breeding values and weighting information for genomic regression analyses. Genetics Selection Evolution, 41(1), Article 55. 10.1186/1297-9686-41-55 PMC2817680 20043827

[tpg220469-bib-0028] Hebbali, A. , & Hebbali, M. A. (2017). Package ‘olsrr’ (Version 0.5, 3) [Computer software]. CRAN.

[tpg220469-bib-0029] Hellwege, J. N. , Keaton, J. M. , Giri, A. , Gao, X. , Velez Edwards, D. R. , & Edwards, T. L. (2017). Population stratification in genetic association studies. Current Protocols in Human Genetics, 95(1), 1.22.1–1.22.23. 10.1002/cphg.48 PMC6007879 29044472

[tpg220469-bib-0030] Henrissat, B. , Heffron, S. E. , Yoder, M. D. , Lietzke, S. E. , & Jurnak, F. (1995). Functional implications of structure‐based sequence alignment of proteins in the extracellular pectate lyase superfamily. Plant Physiology, 107(3), 963–976. 10.1104/pp.107.3.963 7716248 PMC157213

[tpg220469-bib-0031] Hohenfeld, C. S. , Passos, A. R. , de Carvalho, H. W. L. , de Oliveira, S. A. S. , & de Oliveira, E. J. (2022). Genome‐wide association study and selection for field resistance to cassava root rot disease and productive traits. PLoS One, 17(6), e0270020. 10.1371/journal.pone.0270020 35709238 PMC9202857

[tpg220469-bib-0032] Hu, X. , Cheng, L. , Hong, Y. , Li, Z. , Li, C. , & Gu, Z. (2022). Impact of celluloses and pectins restrictions on gluten development and water distribution in potato‐wheat flour dough. International Journal of Biological Macromolecules, 206, 534–542. 10.1016/j.ijbiomac.2022.02.150 35235853

[tpg220469-bib-0033] Huang, M. , Liu, X. , Zhou, Y. , Summers, R. M. , & Zhang, Z. (2019). BLINK: A package for the next level of genome‐wide association studies with both individuals and markers in the millions. GigaScience, 8(2), giy154. 10.1093/gigascience/giy154 30535326 PMC6365300

[tpg220469-bib-0034] Ibe, D. G. , & Ezedinma, F. O. C. (1981). Gari yield from cassava: Is it a function of root yield? In E. R. Terry , K. A. Oduro , & F. Caveness (Eds.), Tropical root crops: Research strategies for the1980s: Proceedings of the first triennial root crops symposium of the International Society for Tropical Root Crops‐Africa Branch (pp. 159–162). International Development Research Centre.

[tpg220469-bib-0035] Ibekwe, U. C. , Chikezie, C. , Obasi, P. C. , & Eze, C. C. (2012). Profitability of garri processing in Owerri north local government area of Imo state. Journal of Science and Technology, 21(4), 343.

[tpg220469-bib-0036] Jaramillo, A. M. , Sierra, S. , Chavarriaga‐Aguirre, P. , Castillo, D. K. , Gkanogiannis, A. , López‐Lavalle, L. A. B. , Arciniegas, J. P. , Sun, T. , Li, L. , & Welsch, R. (2022). Characterization of cassava ORANGE proteins and their capability to increase provitamin A carotenoids accumulation. PLoS One, 17(1), e0262412. 10.1371/journal.pone.0262412 34995328 PMC8741059

[tpg220469-bib-0037] Ji, M. , Fang, W. , Li, W. , Zhao, Y. , Guo, Y. , Wang, W. , Chen, G. , Tian, J. , & Deng, Z. (2021). Genome wide association study of the whiteness and colour related traits of flour and dough sheets in common wheat. Scientific Reports, 11(1), Article 8790. 10.1038/s41598-021-88241-4 33888831 PMC8062544

[tpg220469-bib-0038] Jiang, D. , An, P. , Cui, S. , Sun, S. , Zhang, J. , & Tuo, T. (2020). Effect of modification methods of wheat straw fibers on water absorbency and mechanical properties of wheat straw fiber cement‐based composites. Advances in Materials Science and Engineering, 2020, Article 5031025.

[tpg220469-bib-0039] Johnson, M. , Kumar, A. , Oladzad‐Abbasabadi, A. , Salsman, E. , Aoun, M. , Manthey, F. A. , & Elias, E. M. (2019). Association mapping for 24 traits related to protein content, gluten strength, color, cooking, and milling quality using balanced and unbalanced data in durum wheat [*Triticum turgidum* L. var. Durum (Desf).]. Frontiers in Genetics, 10, 717. 10.3389/fgene.2019.00717 31475032 PMC6706462

[tpg220469-bib-0040] Jones, D. A. , & Jones, J. D. (1997). The role of leucine‐rich repeat proteins in plant defences. In Advances in botanical research (Vol. 24, pp. 89–167). Elsevier.

[tpg220469-bib-0041] Kayondo, S. I. , Pino Del Carpio, D. , Lozano, R. , Ozimati, A. , Wolfe, M. , Baguma, Y. , Gracen, V. , Offei, S. , Ferguson, M. , & Kawuki, R. (2018). Genome‐wide association mapping and genomic prediction for CBSD resistance in Manihot esculenta. Scientific Reports, 8(1), Article 1549. 10.1038/s41598-018-19696-1 29367617 PMC5784162

[tpg220469-bib-0042] Kim, J. , Oh, Y. , Park, C. , Kang, Y. M. , Shin, H. , Kim, I. Y. , & Jang, D. P. (2019). Comparison study of partial least squares regression analysis and principal component analysis in fast‐scan cyclic voltammetry. International Journal of Electrochemical Science, 14(7), 5924–5937. 10.20964/2019.07.03

[tpg220469-bib-0043] Kristensen, P. S. , Jahoor, A. , Andersen, J. R. , Cericola, F. , Orabi, J. , Janss, L. L. , & Jensen, J. (2018). Genome‐wide association studies and comparison of models and cross‐validation strategies for genomic prediction of quality traits in advanced winter wheat breeding lines. Frontiers in Plant Science, 9, Article 69. 10.3389/fpls.2018.00069 29456546 PMC5801407

[tpg220469-bib-0044] Kristensen, P. S. , Jensen, J. , Andersen, J. R. , Guzmán, C. , Orabi, J. , & Jahoor, A. (2019). Genomic prediction and genome‐wide association studies of flour yield and alveograph quality traits using advanced winter wheat breeding material. Genes, 10(9), 669. 10.3390/genes10090669 31480460 PMC6770321

[tpg220469-bib-0045] Kunzek, H. , Kabbert, R. , & Gloyna, D. (1999). Aspects of material science in food processing: Changes in plant cell walls of fruits and vegetables. Zeitschrift Für Lebensmitteluntersuchung Und‐Forschung A, 208, 233–250. 10.1007/s002170050410

[tpg220469-bib-0046] Laya, A. , Koubala, B. B. , Kouninki, H. , & Nukenine, N. E. (2018). Effect of harvest period on the proximate composition and functional and sensory properties of gari produced from local and improved cassava (*Manihot esculenta*) varieties. International Journal of Food Science, 2018, Article 6241035. 10.1155/2018/6241035 29850481 PMC5907523

[tpg220469-bib-0047] Lee, J.‐H. , & Kim, W. T. (2011). Regulation of abiotic stress signal transduction by E3 ubiquitin ligases in Arabidopsis. Molecules and Cells, 31, 201–208. 10.1007/s10059-011-0031-9 21347703 PMC3932693

[tpg220469-bib-0048] Liu, W. , Tang, X. , Qi, X. , Fu, X. , Ghimire, S. , Ma, R. , Li, S. , Zhang, N. , & Si, H. (2020). The ubiquitin conjugating enzyme: An important ubiquitin transfer platform in ubiquitin‐proteasome system. International Journal of Molecular Sciences, 21(8), 2894. 10.3390/ijms21082894 32326224 PMC7215765

[tpg220469-bib-0049] Lou, H. , Zhang, R. , Liu, Y. , Guo, D. , Zhai, S. , Chen, A. , Zhang, Y. , Xie, C. , You, M. , Peng, H. , Liang, R. , Ni, Z. , Sun, Q. , & Li, B. (2021). Genome‐wide association study of six quality‐related traits in common wheat (*Triticum aestivum* L.) under two sowing conditions. Theoretical and Applied Genetics, 134(1), 399–418. 10.1007/s00122-020-03704-y 33155062

[tpg220469-bib-0050] Mbe, J. O. , Dzidzienyo, D. K. , Abah, S. P. , Njoku, D. N. , Onyeka, J. , Tongoona, P. , & Egesi, C. (2024). Novel SNP markers and other stress‐related genomic regions associated with nitrogen use efficiency in cassava. Frontiers in Plant Science, 15, 1376520. 10.3389/fpls.2024.1376520 38638347 PMC11024350

[tpg220469-bib-0051] Merrick, L. F. , & Carter, A. H. (2021). Comparison of genomic selection models for exploring predictive ability of complex traits in breeding programs. The Plant Genome, 14(3), e20158. 10.1002/tpg2.20158 34719886 PMC12807319

[tpg220469-bib-0052] Misra, G. , Badoni, S. , Domingo, C. J. , Cuevas, R. P. O. , Llorente, C. , Mbanjo, E. G. N. , & Sreenivasulu, N. (2018). Deciphering the genetic architecture of cooked rice texture. Frontiers in Plant Science, 9, Article 1405. 10.3389/fpls.2018.01405 30333842 PMC6176215

[tpg220469-bib-0053] Montagnac, J. A. , Davis, C. R. , & Tanumihardjo, S. A. (2009). Nutritional value of cassava for use as a staple food and recent advances for improvement. Comprehensive Reviews in Food Science and Food Safety, 8(3), 181–194. 10.1111/j.1541-4337.2009.00077.x 33467798

[tpg220469-bib-0054] Müller, S. , & Kunzek, H. (1998). Material properties of processed fruit and vegetables I. Effect of extraction and thermal treatment on apple parenchyma: I. Effect of extraction and thermal treatment on apple parenchyma. Zeitschrift Für Lebensmitteluntersuchung Und‐Forschung A, 206, 264–272.

[tpg220469-bib-0055] Nandudu, L. , Kawuki, R. , Ogbonna, A. , Kanaabi, M. , & Jannink, J.‐L. (2023). Genetic dissection of cassava brown streak disease in a genomic selection population. Frontiers in Plant Science, 13, 5627. 10.3389/fpls.2022.1099409 PMC9880483 36714759

[tpg220469-bib-0056] Ndjouenkeu, R. , Ngoualem Kegah, F. , Teeken, B. , Okoye, B. , Madu, T. , Olaosebikan, O. D. , Chijioke, U. , Bello, A. , Oluwaseun Osunbade, A. , Owoade, D. , Takam‐Tchuente, N. H. , Biaton Njeufa, E. , Nguiadem Chomdom, I. L. , Forsythe, L. , Maziya‐Dixon, B. , & Fliedel, G. (2021). From cassava to gari: Mapping of quality characteristics and end‐user preferences in Cameroon and Nigeria. International Journal of Food Science & Technology, 56(3), 1223–1238. 10.1111/ijfs.14790 33776232 PMC7984457

[tpg220469-bib-0057] Neph, S. , Kuehn, M. S. , Reynolds, A. P. , Haugen, E. , Thurman, R. E. , Johnson, A. K. , Rynes, E. , Maurano, M. T. , Vierstra, J. , & Thomas, S. (2012). BEDOPS: High‐performance genomic feature operations. Bioinformatics, 28(14), 1919–1920. 10.1093/bioinformatics/bts277 22576172 PMC3389768

[tpg220469-bib-0058] Ngoualem Kégah, F. , & Ndjouenkeu, R. (2023). Gari, a cassava (*Manihot esculenta* Crantz) derived product: Review on its quality and their determinants. Journal of Food Quality, 2023, Article 7238309.

[tpg220469-bib-0059] Nweke, F. I. (2004). New challenges in the cassava transformation in Nigeria and Ghana (Vol. 118). International Food Policy Research Institute.

[tpg220469-bib-0060] Nyquist, W. E. , & Baker, R. J. (1991). Estimation of heritability and prediction of selection response in plant populations. Critical Reviews in Plant Sciences, 10(3), 235–322. 10.1080/07352689109382313

[tpg220469-bib-0061] Obel, H. O. , Cheng, C. , Li, Y. , Tian, Z. , Njogu, M. K. , Li, J. , Lou, Q. , Yu, X. , Yang, Z. , & Ogweno, J. O. (2022). Genome‐wide identification of the B‐Box gene family and expression analysis suggests their potential role in photoperiod‐mediated β‐carotene accumulation in the endocarp of cucumber (*Cucumis sativus* L.) fruit. Genes, 13(4), 658. 10.3390/genes13040658 35456464 PMC9031713

[tpg220469-bib-0062] Oduro, I. , Ellis, W. O. , Dziedzoave, N. T. , & Nimako‐Yeboah, K. (2000). Quality of gari from selected processing zones in Ghana. Food Control, 11(4), 297–303. 10.1016/S0956-7135(99)00106-1

[tpg220469-bib-0063] Olaosebikan, O. , Abolore, B. , De Sousa, K. , Ndjouenkeu, R. , Adesokan, M. , Alamu, E. , Agbona, A. , Van Etten, J. , Kégah, F. N. , & Dufour, D. (2023). Drivers of consumer acceptability of cassava gari‐eba food products across cultural and environmental settings using the triadic comparison of technologies approach (tricot). Journal of the Science of Food and Agriculture, 104, 4770–4781. 37463325 10.1002/jsfa.12867

[tpg220469-bib-0064] Oluwamukomi, M. O. , & Lawal, O. S. (2020). Textural characteristics of Nigerian foods. In K. Nishinari (Ed.), Textural characteristics of world foods (pp. 361–383). Wiley. 10.1002/9781119430902.ch25

[tpg220469-bib-0065] Oyeyinka, S. A. , Ajayi, O. I. , Gbadebo, C. T. , Kayode, R. M. O. , Karim, O. R. , & Adeloye, A. A. (2019). Physicochemical properties of gari prepared from frozen cassava roots. LWT, 99, 594–599. 10.1016/j.lwt.2018.10.004

[tpg220469-bib-0066] Peprah, B. B. , Parkes, E. , Manu‐Aduening, J. , Kulakow, P. , Van Biljon, A. , & Labuschagne, M. (2020). Genetic variability, stability and heritability for quality and yield characteristics in provitamin A cassava varieties. Euphytica, 216(2), Article 31. 10.1007/s10681-020-2562-7 32055054 PMC6988135

[tpg220469-bib-0067] Phillips, T. P. , Taylor, D. S. , Sanni, L. O. , & Akoroda, M. O. (2004). A cassava industrial revolution in Nigeria: The potential of a new industrial crop . FAO.

[tpg220469-bib-0068] Phumichai, C. , Aiemnaka, P. , Nathaisong, P. , Hunsawattanakul, S. , Fungfoo, P. , Rojanaridpiched, C. , Vichukit, V. , Kongsil, P. , Kittipadakul, P. , & Wannarat, W. (2022). Genome‐wide association mapping and genomic prediction of yield‐related traits and starch pasting properties in cassava. Theoretical and Applied Genetics, 135(1), 145–171. 34661695 10.1007/s00122-021-03956-2

[tpg220469-bib-0069] Piepho, H.‐P. , & Möhring, J. (2007). Computing heritability and selection response from unbalanced plant breeding trials. Genetics, 177(3), 1881–1888. 10.1534/genetics.107.074229 18039886 PMC2147938

[tpg220469-bib-0098] Pradeepkumar, T. , Dijee, B. , Joy, M. , Radhakrishnan, N. V. , & Aipe, K. C. (2001). Genetic variation in tomato for yield and resistance to bacterial wilt. Journal of Tropical Agriculture, 39, 157–158.

[tpg220469-bib-0070] Quinlan, A. R. , & Hall, I. M. (2010). BEDTools: A flexible suite of utilities for comparing genomic features. Bioinformatics, 26(6), 841–842. 10.1093/bioinformatics/btq033 20110278 PMC2832824

[tpg220469-bib-0071] R Core Team . (2020). R: A language and environment for statistical computing. R Foundation for Statistical Computing.

[tpg220469-bib-0072] Rabbi, I. Y. , Udoh, L. I. , Wolfe, M. , Parkes, E. Y. , Gedil, M. A. , Dixon, A. , Ramu, P. , Jannink, J.‐L. , & Kulakow, P. (2017). Genome‐wide association mapping of correlated traits in cassava: Dry matter and total carotenoid content. The Plant Genome, 10(3), plantgenome2016.09.0094. 10.3835/plantgenome2016.09.0094 PMC7822061 29293815

[tpg220469-bib-0073] Salcedo, A. , Zambrana, C. , & Siritunga, D. (2014). Comparative expression analysis of reference genes in field‐grown cassava. Tropical Plant Biology, 7, 53–64. 10.1007/s12042-014-9137-5

[tpg220469-bib-0074] Santner, A. , & Estelle, M. (2010). The ubiquitin‐proteasome system regulates plant hormone signaling. The Plant Journal, 61(6), 1029–1040. 10.1111/j.1365-313X.2010.04112.x 20409276 PMC3066055

[tpg220469-bib-0075] Schüle, T. , Rose, M. , Entian, K.‐D. , Thumm, M. , & Wolf, D. H. (2000). Ubc8p functions in catabolite degradation of fructose‐1, 6‐bisphosphatase in yeast. The EMBO Journal, 19(10), 2161–2167. 10.1093/emboj/19.10.2161 10811607 PMC384366

[tpg220469-bib-0076] Soitamo, A. J. , Piippo, M. , Allahverdiyeva, Y. , Battchikova, N. , & Aro, E.‐M. (2008). Light has a specific role in modulating Arabidopsis gene expression at low temperature. BMC Plant Biology, 8(1), Article 13. 10.1186/1471-2229-8-13 18230142 PMC2253524

[tpg220469-bib-0077] Sun, L. , & van Nocker, S. (2010). Analysis of promoter activity of members of the PECTATE LYASE‐LIKE (PLL) gene family in cell separation in Arabidopsis. BMC Plant Biology, 10, Article 152. 10.1186/1471-2229-10-152 20649977 PMC3017822

[tpg220469-bib-0078] Swissa, M. , Aloni, Y. , Weinhouse, H. , & Benizman, M. (1980). Intermediatry steps in *Acetobacter xylinum* cellulose synthesis: Studies with whole cells and cell‐free preparations of the wild type and a celluloseless mutant. Journal of Bacteriology, 143(3), 1142–1150. 10.1128/jb.143.3.1142-1150.1980 7410313 PMC294464

[tpg220469-bib-0079] Tauberger, E. , Fernie, A. R. , Emmermann, M. , Renz, A. , Kossmann, J. , Willmitzer, L. , & Trethewey, R. N. (2000). Antisense inhibition of plastidial phosphoglucomutase provides compelling evidence that potato tuber amyloplasts import carbon from the cytosol in the form of glucose‐6‐phosphate. The Plant Journal, 23(1), 43–53. 10.1046/j.1365-313x.2000.00783.x 10929100

[tpg220469-bib-0080] Teeken, B. , Agbona, A. , Bello, A. , Olaosebikan, O. , Alamu, E. , Adesokan, M. , Awoyale, W. , Madu, T. , Okoye, B. , & Chijioke, U. (2021). Understanding cassava varietal preferences through pairwise ranking of gari‐eba and fufu prepared by local farmer–processors. International Journal of Food Science & Technology, 56(3), 1258–1277. 33776234 10.1111/ijfs.14862 PMC7984147

[tpg220469-bib-0081] Uchendu, K. , Njoku, D. N. , Paterne, A. , Rabbi, I. Y. , Dzidzienyo, D. , Tongoona, P. , Offei, S. , & Egesi, C. (2021). Genome‐wide association study of root mealiness and other texture‐associated traits in cassava. Frontiers in Plant Science, 12, 770434. 34975953 10.3389/fpls.2021.770434 PMC8719520

[tpg220469-bib-0082] Udoro, E. O. , Kehinde, A. T. , Olasunkanmi, S. G. , & Charles, T. A. (2014). Studies on the physicochemical, functional and sensory properties of gari processed from dried cassava chips. Journal of Food Processing and Technology, 5(1), 293.

[tpg220469-bib-0083] Voragen, A. G. , Coenen, G.‐J. , Verhoef, R. P. , & Schols, H. A. (2009). Pectin, a versatile polysaccharide present in plant cell walls. Structural Chemistry, 20, 263–275. 10.1007/s11224-009-9442-z

[tpg220469-bib-0084] Wallace, J. G. , Bradbury, P. J. , Zhang, N. , Gibon, Y. , Stitt, M. , & Buckler, E. S. (2014). Association mapping across numerous traits reveals patterns of functional variation in maize. PLoS Genetics, 10(12), e1004845. 10.1371/journal.pgen.1004845 25474422 PMC4256217

[tpg220469-bib-0085] Wang, J. , & Zhang, Z. (2021). GAPIT version 3: Boosting power and accuracy for genomic association and prediction. Genomics, Proteomics & Bioinformatics, 19(4), 629–640. 10.1016/j.gpb.2021.08.005 PMC9121400 34492338

[tpg220469-bib-0086] Wang, Z. , Zhao, J. , Guan, Q. , Ke, Z. , Li, X. , Zhang, Z. , Tian, J. , Li, H. , & Chen, J. (2022). QTL analysis for 27 quality traits measured through the color of end‐use products in common wheat (*Triticum aestivum* L.). Euphytica, 218(9), 121.

[tpg220469-bib-0087] Wickham, H. (2016). Getting started with ggplot2. In Ggplot2: Elegant graphics for data analysis (pp. 11–31). Springer.

[tpg220469-bib-0088] Wossen, T. , Girma, G. , Abdoulaye, T. , Rabbi, I. , Olanrewaju, A. , Kulakow, P. , Asumugha, G. , Abass, A. , Tokula, M. , & Manyong, V. (2017). The cassava monitoring survey in Nigeria . IITA.

[tpg220469-bib-0090] Xiong, C. , Luo, D. , Lin, A. , Zhang, C. , Shan, L. , He, P. , Li, B. , Zhang, Q. , Hua, B. , & Yuan, Z. (2019). A tomato B‐box protein Sl BBX 20 modulates carotenoid biosynthesis by directly activating PHYTOENE SYNTHASE 1, and is targeted for 26S proteasome‐mediated degradation. New Phytologist, 221(1), 279–294. 10.1111/nph.15373 30101463

[tpg220469-bib-0091] Yang, J. , Zeng, J. , Goddard, M. E. , Wray, N. R. , & Visscher, P. M. (2017). Concepts, estimation and interpretation of SNP‐based heritability. Nature Genetics, 49(9), 1304–1310. 10.1038/ng.3941 28854176

[tpg220469-bib-0092] Yin, L. (2020). CMplot: Circle Manhattan plot (R package version 3.6) [Computer software]. CRAN.

[tpg220469-bib-0093] Zargar, S. M. , Raatz, B. , Sonah, H. , Muslima, N. , Bhat, J. A. , Dar, Z. A. , Agrawal, G. K. , & Rakwal, R. (2015). Recent advances in molecular marker techniques: Insight into QTL mapping, GWAS and genomic selection in plants. Journal of Crop Science and Biotechnology, 18, 293–308. 10.1007/s12892-015-0037-5

[tpg220469-bib-0094] Zhang, Z. , Ersoz, E. , Lai, C.‐Q. , Todhunter, R. J. , Tiwari, H. K. , Gore, M. A. , Bradbury, P. J. , Yu, J. , Arnett, D. K. , & Ordovas, J. M. (2010). Mixed linear model approach adapted for genome‐wide association studies. Nature Genetics, 42(4), 355–360. 10.1038/ng.546 20208535 PMC2931336

[tpg220469-bib-0095] Zhu, H. , & Zhou, X. (2020). Statistical methods for SNP heritability estimation and partition: A review. Computational and Structural Biotechnology Journal, 18, 1557–1568. 10.1016/j.csbj.2020.06.011 32637052 PMC7330487

